# Breast Cancer knowledge and perceived barriers to help seeking among pre-clinical and clinical female medical students of King Edward Medical University, Lahore: a cross-sectional study

**DOI:** 10.1186/s12909-020-02132-2

**Published:** 2020-07-14

**Authors:** Saleha Qasim, Hamnah Tayyab, Kainat Zulqadar, Somer Masood, Tahira Bano Qasim, Zainab Zubair

**Affiliations:** 1 Bachelor of Medicine and Bachelor of Surgery, King Edward Medical University, Lahore, Pakistan; 2 West Surgical Ward, Department of Surgery, King Edward Medical University, Lahore, Pakistan; 3 Department of Statistics, The Women University Multan, Multan, Pakistan; 4 Surgical Oncology, Shaukat Khanum Memorial Cancer Hospital and Research Centre, Lahore, Pakistan

**Keywords:** Breast cancer, Breast self-examination, Symptoms, Risk factors, Medical students, Barriers, Pakistan

## Abstract

**Background:**

Breast cancer is the most common form of cancer in women and second most common cancer after lung cancer. The prognosis of breast cancer depends on early detection and intervention which in turn relies upon awareness. Health workers in all communities are responsible for educating the population at risk. This study evaluates the knowledge regarding breast cancer, personal judgment of confidence, perceived barriers to help seeking and breast self-examination practices among Pakistani female medical students and studies the impact of clinical training on the studied variables.

**Methods:**

Descriptive cross-sectional study design using self-administered validated questionnaires (BCAM-Breast Cancer Awareness Measure designed by Cancer Research UK) was employed. Female medical students enrolled in clinical and pre-clinical classes of King Edward Medical University, Lahore were targeted and questioned regarding symptoms, risk factors and their practices regarding breast cancer. Possible barriers to seeking help were also studied.

**Result:**

The mean number of symptoms correctly identified was 6.7 ± 3.2 (5.5 ± 3.2 for pre-clinical and 7.8 ± 2.9 for clinical students) and for risk factors it was 4.3 ± 2.1 (3.7 ± 2.1 for pre-clinical and 4.7 ± 2.2 for clinical students). The difference in the level of perception of two groups was found to be significant (*p* < 0.001 for symptoms and p < 0.001 for risk factors). 38.7% of the subjects responded that they check their breasts rarely, 33.1% were fairly confident while 8.6% were very confident about detecting a change in their breast, 50.0% never noticed a change in their breast, and 77.4% will contact a doctor within a week or less of finding a change in their breast. Confidence about detecting a change significantly improved (*p* < 0.001) after the start of clinical training.

**Conclusion:**

This study suggests that clinical training may have improved knowledge of female medical students regarding breast cancer; yet the knowledge related to the symptoms and risk factors of breast cancer and frequency of breast self-examination of female medical students is less than anticipated.

## Background

Breast cancer is the most common form of cancer in women and second most common cancer after lung cancer. According to GLOBOCAN 2012, 1.67 million cases of breast cancer were diagnosed in 2012. It was responsible for 522,000 deaths that year and the most frequent cause of cancer death in women in less developed regions [1].

Pakistan has the highest prevalence of breast cancer in Asia with one in every nine women suffering from the disease [2, 3]. A study on the incidence and mortality of breast cancer demonstrated that the highest age standardized death rate (2.52) in Asia due to breast cancer was observed in Pakistan [4]. The frequency of breast cancer in Karachi was reported to be 69.1 per 100,000 from 1998 to 2002 [5].

The risk factors for the disease vary widely and include age, weight, family history, nutrition, general and reproductive health, age at menarche and menopause, smoking, alcohol and lifestyle [6]. The dangerous complications of breast cancer can be reduced significantly if it is detected early. Early detection depends on knowing signs and symptoms of breast cancer and detection methods such as breast self-examination, mammography, and clinical breast examination. The survival rates for the disease vary greatly worldwide, ranging from 80% or over in North America, Sweden and Japan to around 60% in middle-income countries and below 40% in low-income countries. The difference is likely due to high awareness levels leading to early detection and treatment in developed countries [7]. Doctors and health care professionals play a central role in the proper education of the patients and general population. Hence, the knowledge of medical students regarding breast cancer is a crucial factor.

There is no significant data available regarding the impact of clinical exposure on Pakistani female medical students’ knowledge of breast cancer. Sambanje conducted a cross sectional survey on the knowledge of pre-medical students of Angola and suggested that it would be interesting to investigate how knowledgeable the same students would be after their clinical courses [8].

Clinical learning is the essence of medical education. Generally, it is presumed that after the commencement of clinical years of medical training, the exposure of students to the common public health issues and the diseases with higher incidence in the community is increased. Since the early times of medical education, clinical exposure has been considered a crucial part of medical curriculum and the centers with the best clinical opportunities are regarded as the best centers of medical education. Given the prime importance attributed to clinical exposure and training, it is essential that it must be evaluated from time to time to ensure quality. To evaluate the impact of clinical exposure on female students at King Edward Medical University Lahore related to breast cancer education, this study addressed the question ‘How does clinical exposure affect knowledge, confidence and practices of students regarding breast cancer?’

Previous studies have shown that there exists a difference between knowledge of students related to various diseases before and after their exposure to clinical settings. A study in Hong Kong showed that senior medical students had better overall knowledge about organ donation and that there was a positive correlation between knowledge and seniority [9]. The results of a study in four Asian countries revealed that final year dental and dental hygiene students were better aware of the relationship between smoking and diseases compared to entry-level students [10]. However, there was insufficient data comparing the knowledge or attitude of medical students regarding breast cancer before and after clinical training in Pakistan. In a study conducted in United States, breast cancer knowledge was measured upon entry to and exit from medical school and it was found that although the knowledge improved during training, there was still room for improvement [11]. In another study, association of medical training with knowledge about breast cancer and clinical breast examination (CBE) skills was measured and it was observed that although knowledge about breast cancer improved, clinical breast exam skills declined with higher level of training [12].

This study was designed with the objectives of assessing the knowledge of female medical students in their preclinical and clinical phases of professional training about the symptoms and risk factors of breast cancer, their perceived barriers of seeking help, self-reported confidence to detect any change in the breast and their breast self-examination practices.

## Methods

### Participants and method

A cross-sectional survey was carried out to assess the general level of awareness as well as the effect of clinical exposure on the knowledge of female medical students regarding breast cancer. The research proposal was approved from the Institutional Review Board of King Edward Medical University Lahore, Pakistan. Taking into view the objectives of the study, female students, in the second and third years of undergraduate medical training were targeted by distributing questionnaires after lectures and sharing online questionnaire in class groups. All female medical students in 2nd and 3rd year were approached according to the best of knowledge of the authors. According to the teaching system and curriculum as per the requirements of Pakistan Medical and Dental Council (PMDC), the basic theoretical section on breast cancer is included in the curriculum of first year along with thorax in anatomy; second year only contains the embryological development of mammary glands (which was not assessed in the study), the history (along-with symptoms and risk factors), examination and clinical aspects of breast cancer are taught in wards during clinical rotations, fourth year syllabus contains pathological types and risk factors, while final year includes staging and treatment details (PMDC revised curriculum 2018) [13]. Clinical training starts from the third year of program. Hence, the sample had two groups of students. Those from second year had completed 4 months of 2nd year training; but with no clinical exposure. They studied basic subjects of anatomy, physiology and biochemistry with clinical correlation. Those from 3rd year were recently exposed to clinics and had 4 months of preliminary training in history taking and clinical examination.

### Questionnaire

Participants completed a self-administered questionnaire. The questionnaire used was a modified version of Breast Cancer Awareness Measure (BCAM) developed by Cancer Research UK, King’s College London and University College London in 2009. It was validated with the support of Breast Cancer Care and Breakthrough Breast Cancer [14]. The questionnaire has two parts; the first examines knowledge of the symptoms of breast cancer, knowledge of risk factors, confidence to check any change in the breast and the probable reasons for delaying visit to the doctor after noticing a change in breast; the second records the demographic characteristics of the respondent. A brief description of the study was included at the top of the questionnaire in the consent form guaranteeing confidentiality. A total of 266 students completed the questionnaire: 138 from 3rd year/1st clinical year and 128 from 2nd year/pre-clinical year. The total number of enrolled female students in the 2nd year was 179 and in the 3rd year was 191; hence the response rate was 71.5% for 2nd year, 72.3% for 3rd year with an overall response rate of 71.9%.

### Analysis

Mean number of symptoms and risk factors identified by each group as well as the whole of sample were calculated and the association between clinical exposure and breast cancer awareness was observed by using two-sided Pearson’s chi square test. As both the year of study (pre-clinical or clinical) and the ability to identify a given symptom or risk factor were categorical variables, to determine their association Pearson’s chi-square test was employed. Similarly, the association of year of study with responses to other questions (all multiple-choice questions) was also studied using chi-square test. A t-test was employed to compare the mean number of symptoms and risk factors identified by the two groups. *P*-value< 0.05 was considered as significant. The demographics of the two groups were compared to check the similarity of the two samples.

## Results

### Demographic characteristics

The demographic characteristics of each group (i.e. clinical and pre-clinical medical students) as well as of the whole sample are given in Table 1. A majority of the participants of the study were between the ages of 18–21 years (mean age 19.3 ± 3.8), living in university hostels (71.1%), born in Pakistan (97.7%) and Urdu was the main language spoken in their homes (82%). There were no significant difference in the demographic characteristics of the two groups.

**Table 1:** Demographic characteristics of participants

VARIABLE	Pre-clinical Medical Students (***n*** = 128)	Clinical Medical Students (***n*** = 138)	Total (***n*** = 266)
**Mean Age**	18.4 (±4.5)	20.2 (±2.6)	19.3 (±3.8)
**Boarder**	86 (67.2%)	103 (74.6%)	189 (71.1%)
**Country of Birth**			
Pakistan	126 (98.4%)	134 (97.1%)	260 (97.7%)
**Family’s Living Arrangements:**			
Live in Self owned House	96 (75.0%)	114 (83.2%)	210 (79.2%)
House on Rent	15 (11.7%)	11 (8.0%)	26 (9.8%)
House Provided by Employer	7 (5.5%)	9 (6.6%)	16 (6.0%)
**Live with**			
Parents and Siblings	107 (83.6%)	125 (91.2%)	232 (87.5%)
Joint Family	13 (10.2%)	9 (6.6%)	22 (8.3%)
**Somebody at home owns a car or van**	106 (82.8%)	123 (89.1%)	229 (86.1%)
**Ethnicity:**			
Punjabi	85 (66.4%)	74 (53.6%)	159 (59.8%)
Seraiki	11 (8.4%)	13 (9.4%)	24 (9.0%)
Sindhi	13 (10.2%)	35 (25.4%)	48 (18.0%)
Pathan	4 (3.1%)	3 (2.2%)	7 (2.6%)
Other	6 (4.7%)	6 (4.3%)	12 (4.5%)
**Family’s Major Source of Income:**			
Wages or Salary	90 (70.3%)	104 (75.4%)	194 (72.9%)
Business	32 (25%)	31 (22.5%)	63 (23.7%)

To assess the socioeconomic background of the respondents following questions were asked: what is the major source of income of the family, whether the family owns or rents a house, if someone in the immediate family owns a car or van, and whether they have an extended family living together or a nuclear family with only parents and siblings living in the house. These factors were taken into consideration as nuclear family system is prevalent in Pakistan and majority of the female students are financially supported by their parents or guardians till they complete their education.

### Knowledge about symptoms of breast cancer

Table 2 summarizes the knowledge of the respondents about ten symptoms of breast cancer. The questionnaire contained a list of ten symptoms and the respondents were asked to identify the symptoms of breast cancer in the list. All of the symptoms mentioned in the list were indeed actual symptoms. A mean of 6.7 ± 3.2 were correctly identified by the respondents; 5.5 ± 3.2 for 2nd/pre-clinical year and 7.8 ± 2.9 for 3rd/clinical year. A statistically significant difference was found between the two groups for nine out of ten symptoms with the clinical students having a better knowledge.

**Table 2:** Knowledge about symptoms of breast cancer

Symptom of Breast Cancer	Pre-clinical Medical Students (***n*** = 128)	Clinical Medical Students (***n*** = 138)	Total Students (***n*** = 266)	chi square	***p***-value
	Number (%) of respondents who correctly identified the symptom				
Change in position of nipple	68 (53.1%)	120 (87.0%)	188 (70.7%)	36.674	< 0.001
Pulling in of your nipple	62 (48.4%)	108 (78.3%)	170 (63.9%)	25.607	< 0.001
Pain in one of your breasts or armpit	79 (61.7%)	110 (79.7%)	189 (71.1%)	10.451	0.001
Puckering or dimpling of your breast skin	92 (71.9%)	119 (86.2%)	211 (79.3%)	8.345	0.003
Discharge or bleeding from your nipple	46 (35.9%)	104 (75.4%)	150 (56.4%)	41.972	< 0.001
A lump or thickening in your breast	102 (79.7%)	123 (89.1%)	225 (84.6%)	4.542	0.025
Nipple rash	39 (30.5%)	65 (47.1%)	104 (39.1%)	7.715	0.004
Redness of your breast skin	78 (60.9%)	96 (69.6%)	174 (65.4%)	2.185	0.089
A lump or thickening under your armpit	66 (51.6%)	110 (79.7%)	176 (66.2%)	23.502	< 0.001
Changes in the shape of your breast or nipple	72 (56.2%)	115 (83.3%)	187 (70.3%)	23.33	< 0.001
**Mean number of symptoms identified (M ± SD)**	**5.5 ± 3.1**	**7.8 ± 2.9**	**6.7 ± 3.2**		

An independent sample t-test was applied to compare the means of two groups. There was a statistically significant difference in the mean number of symptoms identified by the pre-clinical (M = 5.5, SD = 3.2) and clinical (M = 7.8, SD = 2.9) with t (264) = 6.10; *p* < 0.001.

### Knowledge about risk factors of breast Cancer

Risk factor knowledge was assessed in the same way as the symptoms; i.e., by giving a list of ten risk factors and asking the respondents to identify the risk factors of breast cancer. Again, all the risk factors mentioned in the list were actual risk factors. The mean number of risk factors correctly identified by the pre-clinical/2nd year students was 3.7 ± 2.1 and in the clinical/3rd year students was 4.7 ± 2.2 with a collective mean of 4.3 ± 2.1 as shown in Table 3.

**Table 3:** Knowledge about risk factors of breast cancer

Risk Factors	Pre-clinical Medical Students (***n*** = 128)	Clinical Medical Students (***n*** = 138)	Total Students (***n*** = 266)	chi square	***p***-value
	Number (%) of respondents who correctly identified the risk factor				
Age	4 (3.1%)	17 (12.3%)	21 (7.9%)	7.719	0.004
Having a history of breast cancer	104 (81.2%)	124 (89.9%)	228 (85.7%)	4.016	0.033
Using HRT (Hormone Replacement Therapy)	51 (39.8%)	84 (61.3%)	135 (50.9%)	12.206	< 0.001
Drinking more than 1 unit of alcohol a day	45 (35.2%)	52 (37.7%)	97 (36.5%)	0.183	0.382
Being overweight (BMI over 25)	48 (37.5%)	41 (29.7%)	89 (33.5%)	0.195	0.112
Having a close relative with breast cancer	67 (52.3%)	99 (71.7%)	166 (62.4%)	10.648	0.001
Having children later in life or not at all	37 (28.9%)	67 (48.6%)	104 (39.1%)	10.762	0.001
Starting your periods at an early age	19 (14.8%)	28 (20.3%)	47 (17.7%)	1.354	0.158
Having a late menopause	30 (23.4%)	41 (29.7%)	71 (26.7%)	1.335	0.155
Doing less than 30 mins of moderate physical activity 5 times a week	43 (33.6%)	37 (26.8%)	80 (30.1%)	1.452	0.142
**Mean number of risk factors identified**	3.7 ± 2.1	4.7 ± 2.2	4.3 ± 2.1		

An independent sample t-test was applied to compare the means of the two groups. There was a statistically significant difference in the mean number of risk factors identified by the pre-clinical (M = 3.7, SD = 2.1) and clinical (M = 4.7, SD = 2.2) students with t (264) = 3.79; *p* < 0.001.

### Breast examination practices

Table 4 summarizes the participant responses related to the breast examination practices and history of any noticeable change in breast. No significant differences in the frequency of breast self-examination (BSE) were found between second and third year students (*p* = 0.890).

**Table 4:** Breast examination practices and history of seeing a doctor for a change in breast

Question	Pre-clinical Medical Students (***n*** = 128)	Clinical Medical Students (***n*** = 138)	Total Students (***n*** = 266)
**How often do you check your breasts?**			
rarely or never	52 (40.6%)	51 (37.0%)	103 (38.7%)
once every six months	14 (10.9%)	17 (12.3%)	31 (11.7%)
at least once a month	30 (23.4%)	34 (24.6%)	64 (24.1%)
at least once a week	29 (22.7%)	30 (21.7%)	59 (22.2%)
Total	128 (100%)	137 (99.3%)	265 (99.6%)
***Chi square***	***χ***^**2**^***=1.693***	***p = 0.890***	
**Have you ever been to see a doctor about a change you have noticed in one of your breasts?**			
Yes	9 (7.0%)	12 (8.7%)	21 (7.9%)
no	62 (48.4%)	50 (36.2%)	112 (42.1%)
never noticed a change in my breasts	57 (44.5%)	76 (55.1%)	133 (50.0%)
Total	128 (100%)	138 (100%)	266 (100%)
***Chi square***	***χ***^**2**^***=4.058***	***p = 0.131***	

### Anticipated delay in help seeking

No significant differences were found for the anticipated delay in help seeking (*p* = 0.104) where most of the students responded that they will contact a doctor within a week or less (74.2% from 2nd year, 80.5% from 3rd year and 77.4% of the total respondents).

### Confidence to detect a change in breast

Table 5 shows the difference in the level of confidence to detect any change in the breast which was observed to be significantly higher among the 3rd year/clinical medical students compared to 2nd year/pre-clinical medical students (*p* < 0.001). 33.1% of the participants were fairly confident and 8.6% were very confident to notice any change in their breasts.

**Table 5:** Confidence to detect any change in the breast

Are you confident you would notice a change in your breasts?			
not at all confident	45 (35.2%)	22 (15.9%)	67 (25.2%)
slightly confident	47 (36.7%)	41 (29.7%)	88 (33.1%)
fairly confident	27 (21.1%)	61 (44.2%)	88 (33.1%)
very confident	9 (7.0%)	14 (10.1%)	23 (8.6%)
Total	128 (100%)	138 (100%)	266 (100%)
***Chi square***	***χ***^**2**^***=22.183***	***p*** < 0.001	

### Barriers to help seeking

The participants were asked whether they would delay going to the doctor due to a series of emotional, practical or service barriers (Table 6). They were provided with a list of potential barriers to choose. The most frequently identified reasons for not going to the doctor in time were: too busy to go to the doctor (58.0%) followed by having too many other things to worry about (50.6%) and embarrassment (49.4%). Only 8.8% of the students were worried about wasting doctors’ time.

**Table 6:** Barriers to seeking help after finding a possible symptom of breast cancer

Could you say if any of these reasons might put you off going to the doctor?	Number (%) of students who responded as sometimes yes or often yes			chi square	***p***-value
	Pre-clinical Medical Students (***n*** = 128)	Clinical Medical Students (***n*** = 138)	Total Students (***n*** = 266)		
Are you too embarrassed to go and see the doctor?	66 (53.2%)	63 (46.0%)	129 (49.4%)	1.365	0.148
Are you too scared to go and see the doctor?	51 (40.5%)	49 (35.5%)	100 (37.9%)	0.691	0.241
Are you worried about wasting the doctor’s time?	13 (10.7%)	10 (7.2%)	23 (8.8%)	0.933	0.227
Do you find your doctor difficult to talk to?	41 (33.1%)	41 (29.9%)	82 (31.4%)	0.297	0.340
Is it difficult for you to make an appointment with the doctor?	30 (23.4%)	37 (26.8%)	67 (25.2%)	0.401	0.312
Are you too busy to make time to go to the doctor?	74 (59.7%)	78 (56.5%)	152 (58.0%)	0.267	0.348
Do you have too many other things to worry about than going to the doctor?	69 (55.2%)	64 (46.4%)	133 (50.6%)	2.043	0.096
Is it difficult for you to arrange transport to the doctor’s surgery?	16 (13.0%)	19 (13.8%)	35 (13.4%)	0.032	0.502
Are you afraid that worrying about what the doctor might find may stop you from going to the doctor?	44 (35.5%)	35 (25.4%)	79 (30.2%)	3.177	0.050
Not feeling confident talking about my symptom with the doctor	62 (50.0%)	53 (38.4%)	115 (43.9%)	3.565	0.039

The response to following barriers was significantly different between the pre-clinical and clinical students: not feeling confident talking about the symptom with the doctor (*p* = 0.039) and fear of worrying about what the doctor might find (*p* = 0.050). There were no significant differences for other barriers.

## Discussion

This study suggests a statistically significant difference between female medical students before and after clinical exposure regarding the knowledge of symptoms and risk factors of breast cancer and confidence to notice the warning signs, with the clinical students being more aware about breast cancer. The results are in agreement with studies done on undergraduate university students and healthcare professionals in Pakistan and other parts of the world [7, 8, 15–20].

Knowledge of breast cancer symptoms and risk factors is essential for seeking health services. Studies targeting the general population suggest knowledge related to specific symptoms and risk factors is lacking. An analytical study based on data from national surveys in the UK showed that only 1% of women (all ages) were able to identify that the risk of breast cancer is greatest at advanced ages [21]. Similarly, studies conducted among high school and undergraduate students worldwide have shown the inadequacy of knowledge of common breast cancer risk factors [8, 16, 22]. Moreover, limited awareness regarding cancer and its risk factors has been shown in various other cross sectional studies [23–28]. Many of the studies reviewed employed the same questionnaire used in this study, as it is a validated questionnaire developed by a reputable organization (Cancer Research UK). Unfortunately, this study’s results are no different from those conducted on the general population indicating that the knowledge of medical students regarding risk factors of breast cancer was comparable to non-professionals [29, 30]. In a study conducted on females (non-healthcare personals) in Bahawalpur, Pakistan, 42.6% of participants identified HRT as a risk factor for breast cancer, 40.7% identified obesity, 27.9% childbirth at age above 30 years and 20.3% identified late menopause [29]. Whereas in our study, 39.8% of pre-clinical students identified HRT, 37.5% obesity, 28.9% childbirth later in life and 23.4% identified late menopause. Moreover, figures from the total participants of the current study were also comparable. This suggests that both studies yielded similar results when it came to knowledge regarding breast cancer risk factors.

Students with clinical training did not report performing of breast self-examination (BSE) more frequently. Only 24.1% of the students performed BSE every month. This is in contrast to a study conducted on female health care professionals in a tertiary institution in Lagos, Nigeria in which 95% female health care workers performed BSE every month [31]. However, results similar to this study have been reported in Iran and Saudi Arabia where only 6 and 17.3% of the health care professionals performed BSE monthly [32]. Since physicians and other healthcare workers ought to be the role models and promoters for early detection, there is a dire need of interventions to improve the attitude of medical students and other health care workers.

Confidence to detect any change in one’s breast and to talk about that symptom with a doctor is important in early detection. In a study conducted in the Gaza strip, 13.8% women were fairly confident and 12.4% were very confident of noticing any change in their breasts [33]. Similarly, a study in Saudi Arabia reports that 18.7% of their female participants were confident to notice any breast tumor on BSE [34]. The overall confidence level of the medical students in our study was remarkably high and higher for those commencing clinical training.

The possible predictors of any anticipated delay in contacting the doctor after noticing a change were numerous; being busy, being worried about other things and embarrassment were few of the top reasons provided by students. These results point to possible shortcomings of the health system, as they show that health may not be a priority of the respondents and going to the doctor might be considered a time consuming task. Around 50% of the respondents were embarrassed to go to the doctor, indicating social stigmas needing to be addressed. Moreover, it is also possible that the environment in which the patients are examined may not be adequately comfortable or ensure complete privacy. Similar results were shown in studies conducted in other countries [26]. Furthermore, 49.4% of the respondents were embarrassed to go to the doctor compared to 30% who were afraid of worrying about what the doctor might find. This indicates that embarrassment is a greater factor than fear of being diagnosed with cancer that would keep participants from seeking help. Another 37.9% were scared to see a doctor and 31.4% found it difficult to talk to the doctor. These figures show that hesitancy and communication barriers may also delay help seeking. Similar results for reasons of delay in seeking help, including shyness (37.0%) and unavailability of female doctors (15.5%), were reported in a study conducted on females visiting outpatient departments (OPD) at Pak Emirates Military Hospital Rawalpindi, Pakistan [30]. Two of the reasons for delaying a visit to the doctor were significantly more frequently (*p* = .05 and *p* = .039) chosen by the pre-clinical students. These were: being afraid of what the doctor might find and not feeling confident about talking to the doctor. It shows that with the passing time and continued training, fear may subside. No significant difference was found between the two groups regarding other reasons for delay in contacting a doctor, indicating those having clinical training had no greater motivation to seek care.

The scope of this study is limited as it targeted the students of only one medical institute, and therefore might not be a representative of all medical institutes across Pakistan. Moreover, the study was conducted on students of 3rd year who were only beginning clinical experience and the overall adequacy of clinical training could not be assessed. Additionally, this study involves only the female medical students as the questionnaire employed was only validated for females. The design of questionnaire poses another limitation as all the symptoms and risk factors mentioned in the lists were the actual symptoms and risk factors of breast cancer. The students might have thought that some of the enlisted options were not correct and refrained from choosing them all.

Future research is necessary to study other factors, such as the relation of social class, income, education of parents and the area of residence with the students’ knowledge and attitude about breast cancer. Furthermore, studies must be conducted that analyze the curriculum and clinical training of medical students in Pakistan to identify what learning experiences have greatest impact.

Although the study strongly suggests that students with clinical exposure have higher knowledge and confidence concerning monitoring and detecting breast cancer, it also indicates the overall inadequacy of knowledge.

## Conclusions

The knowledge of symptoms and risk factors of breast cancer improved with clinical exposure of students and they also showed significant increase in confidence to notice symptoms of breast cancer. However, most of the perceived barriers to seeking help did not change significantly. Despite the improvement, the level of knowledge among the participants was comparable to non-healthcare personals shown in various other studies [29, 30]. This indicates a need for a considerable improvement.

## Data Availability

The datasets used and/or analyzed during the current study are available from the corresponding author on reasonable request.

## Abbreviations

BC: Breast Cancer
KEMU: King Edward Medical University
PMDC: Pakistan Medical and Dental Council
IRB: Institutional Review Board
